# Preventive Strategies for Pediatric Health in Primary Healthcare: A Systematic Review

**DOI:** 10.7759/cureus.78719

**Published:** 2025-02-07

**Authors:** Khadijah M Bohaligah, Maryam M Bohaligah, Sarah M Bohaligah

**Affiliations:** 1 Pediatric Emergency, Maternity and Children Hospital, Dammam, SAU; 2 Pediatrics, Sheikh Shakhbout Medical City (SSMC), Abu Dhabi, ARE; 3 General Practice, Imam Abdulrahman Bin Faisal University, Dammam, SAU

**Keywords:** developmental screening, immunization, nutrition counseling, parental education, pediatric preventive care

## Abstract

This systematic review evaluates the effectiveness of preventive strategies in pediatric primary care, focusing on immunization, developmental screening, nutrition counseling, physical activity programs, accident prevention, mental health screening, oral health programs, and parental education, which are essential for enhancing child health outcomes and preventing long-term health issues. Following the Preferred Reporting Items for Systematic Reviews and Meta-Analyses (PRISMA) guidelines, a comprehensive literature search was conducted across multiple databases, including PubMed, MEDLINE, EMBASE, Cochrane Library, and PsycINFO, covering publications from January 2000 to December 2023, with 20 studies included encompassing randomized controlled trials (RCTs), cohort studies, observational studies, and systematic reviews. The review demonstrated that immunization programs significantly reduced disease incidence, while developmental screenings allowed for early intervention in developmental delays. Nutrition counseling and physical activity programs effectively addressed childhood obesity, safety education during pediatric visits reduced injury rates, and mental health screenings facilitated early detection of psychological issues, although with some variability in outcomes. Oral health programs improved dental outcomes, and parental education enhanced the effectiveness of preventive strategies, albeit with varying degrees of success depending on socioeconomic contexts. Incorporating preventive strategies into pediatric care is key to better child health, and providers need support to overcome implementation challenges. Further research is needed to optimize these strategies and evaluate their long-term impact across diverse populations and settings.

## Introduction and background

Pediatric health is a cornerstone of primary care, with preventive strategies essential for ensuring healthy development and averting disease. Although extensive literature exists on pediatric preventive care, full integration of these strategies in primary care remains limited due to resource constraints, training deficiencies, time pressures [1,2], and variability in guideline adherence [3,4].

This systematic review evaluates the effectiveness of preventive strategies, including pediatric immunization, developmental screening, nutrition counseling, physical activity programs, accident prevention, mental health screening, oral health programs, and parental education, in primary care settings. By synthesizing current evidence, the review aims to provide a consolidated framework for effective preventive practices. Previous research demonstrates that integrating these strategies improves health outcomes and reduces the incidence of preventable diseases [5,6]. However, while immunization and developmental screening are well supported, further research on mental health screening and parental education is needed [7,8].

## Review

Methodology

Study Design

This systematic review follows the Preferred Reporting Items for Systematic Reviews and Meta-Analyses (PRISMA) guidelines. We aimed to evaluate and synthesize existing research on preventive strategies in pediatric primary care, including immunization, developmental screening, nutrition counseling, physical activity programs, accident prevention, mental health screening, oral health programs, and parental education.

Search Strategy

We conducted a comprehensive literature search across multiple databases, including PubMed, MEDLINE, EMBASE, Cochrane Library, and PsycINFO. We included articles published from January 2000 to December 2023 in our search. The keywords used in the search included "pediatric preventive care," "primary care," "immunization," "developmental screening," "nutrition counseling," "physical activity," "accident prevention," "mental health screening," "oral health," and "parental education." Boolean operators and Medical Subject Headings (MeSH) terms were utilized to refine the search.

Inclusion and Exclusion Criteria

The inclusion criteria for this review consist of studies published in peer-reviewed journals that investigate preventive strategies within pediatric primary care settings. Eligible study designs include randomized controlled trials (RCTs), cohort studies, observational studies, and systematic reviews, provided they are published in English. On the other hand, studies focusing on non-primary care settings or those unrelated to preventive strategies in pediatrics will be excluded. Additionally, editorials, commentaries, case reports, and non-English publications will not be considered for inclusion.

Study Selection

We initially found a total of 1,200 articles. After removing duplicates, 1,050 articles remained. Two independent reviewers screened the titles and abstracts against the inclusion and exclusion criteria. Discrepancies were resolved through discussion or consultation with a third reviewer. A total of 200 articles were selected for full-text review. Finally, 20 studies met the criteria for inclusion in this systematic review.

Data Extraction

Data were extracted using a standardized form designed to capture comprehensive information from each study. Two independent reviewers conducted the data extraction to ensure accuracy and minimize bias. The form recorded study characteristics such as the author, publication year, journal, and study design. In addition, it included population details such as age range, sample size, and setting. Preventive strategies evaluated in each study, along with their associated outcome measures and key findings, were also systematically documented. Any discrepancies between the reviewers were resolved through discussion, and if necessary, a third reviewer was consulted to reach a consensus.

Quality Assessment

We assessed the quality of the included studies using the Cochrane Risk of Bias Tool for randomized controlled trials and the Newcastle-Ottawa Scale for observational studies. Two independent reviewers performed the assessments, rating studies as having low, moderate, or high risk of bias. Any discrepancies between the reviewers were resolved by consensus, and if agreement could not be reached, a third reviewer was consulted. Additionally, we employed the Grading of Recommendations, Assessment, Development, and Evaluation (GRADE) approach to determine the overall certainty of the evidence by considering study design, risk of bias, inconsistency, imprecision, and publication bias.

Data Synthesis

We conducted a narrative synthesis of the findings. Studies were grouped based on the preventive strategy assessed. The effectiveness of each strategy was summarized, highlighting key outcomes and implications for practice. When reported data were incomplete, we contacted study authors (when feasible) for clarification. If missing data could not be retrieved, we performed descriptive analysis without imputations.

PRISMA Flow Diagram

**Figure 1:**
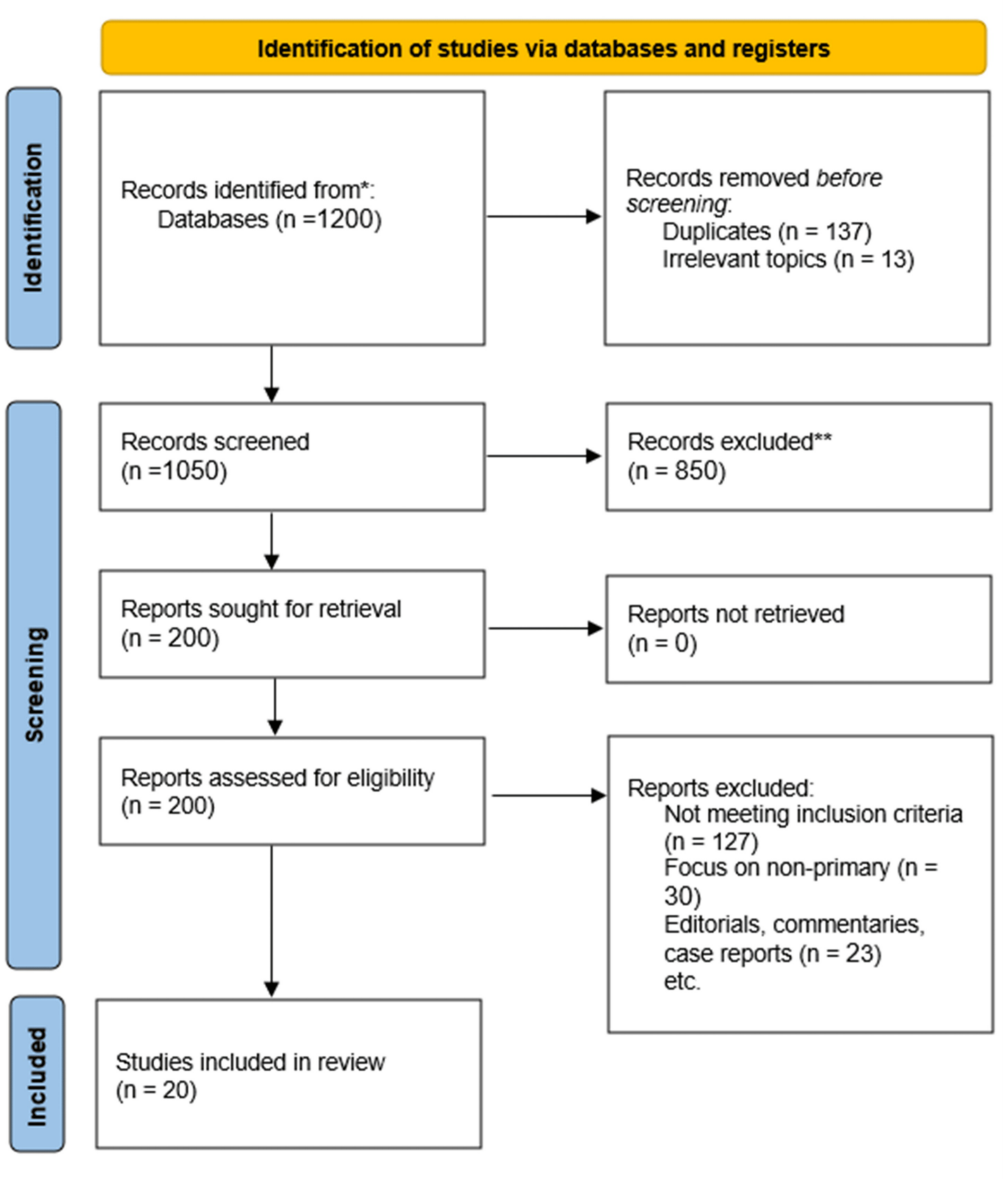
PRISMA Flow Diagram PRISMA: Preferred Reporting Items for Systematic Reviews and Meta-Analyses

The following PRISMA flow diagram illustrates the study selection process (Figure 1).

Results

This section presents the findings from the 20 studies included in the systematic review, organized by preventive strategy.

Multi-component Interventions

Alexander et al. (2017) conducted a systematic review in the USA, finding that multi-component interventions significantly improved the delivery of preventive services in primary care for preschool children [1]. They reviewed 29 studies from the United States, finding that multi-component interventions, combining training for health practitioners and office staff with modifications to the physical environment and practice support, may be more effective than single-component approaches. The review also stressed the need for high-quality studies incorporating clinical endpoints to clearly demonstrate health benefits for children.

Immunization

Gavagan et al. (2010) conducted a retrospective review in the USA, evaluating a pay-for-performance program in a network of publicly funded primary care clinics [9]. Their intervention focused on improving several preventive services, including cervical cancer screening, mammography, and pediatric immunizations. Although some performance indicators improved over time, there were no clinically significant differences between clinics with financial incentives and those without. Most physicians surveyed felt the incentives were not very effective in improving quality of care, suggesting that the magnitude and structure of the incentives may have limited their impact.

Developmental Screening

Developmental screening is crucial for the early identification of developmental delays. Perrin et al. (2016) conducted an observational study in the USA, reporting that routine developmental screenings allowed for timely interventions, leading to improved cognitive and behavioral outcomes [7]. Their findings indicated that early detection of motor, linguistic, mental, or social development issues often led to effective early interventions. The Committee on Practice and Ambulatory Medicine (2000) emphasized the importance of continuous developmental monitoring during well-child visits, which ensures early detection and management of developmental issues [4]. They noted that motor development at 90 days was correlated with motor development at 57 months, highlighting the predictive value of early screenings.

Nutrition Counseling

Nutrition counseling emerged as a vital component of pediatric preventive care. Gorin et al. (2014) conducted an RCT in the USA, demonstrating that structured nutrition counseling during routine visits effectively reduced the prevalence of childhood obesity [5]. Their intervention included brief motivational counseling (BMC) by primary care clinicians and monthly contact with community health workers (CHWs), which significantly reduced obesogenic behaviors in high-risk children.

Brown and Perrin (2018) highlighted the importance of primary care providers in promoting healthy eating habits among children and their families [2]. Their systematic review emphasized early intervention from infancy, focusing on healthy feeding, activity, and family lifestyle behaviors. They outlined a four-stage treatment model for childhood obesity, underscoring the need for comprehensive, evidence-based practices to support healthy growth trajectories.

Physical Activity Programs

Physical activity programs play a crucial role in preventing obesity and promoting overall health. Vine et al. (2013) reviewed various interventions and concluded that integrating physical activity counseling into primary care visits was effective in encouraging children to adopt active lifestyles [3]. They found that primary care providers could promote physical activity through clinical and community settings, emphasizing the need for weight status assessment, healthy lifestyle promotion, and community program referrals. Sherwood et al. (2013) reported significant improvements in physical activity levels among children at risk for obesity following primary care-based interventions [10]. Their study combined brief counseling during well-child visits with follow-up telephone coaching to support healthful eating and activity patterns, demonstrating the effectiveness of such integrated approaches.

Accident Prevention

Accident prevention is a critical component of pediatric preventive care, aimed at reducing injury rates among children. Section on Pediatric Dentistry and Oral Health (2008) conducted an RCT in the USA, finding that safety education during pediatric visits, such as proper use of car seats and home safety measures, significantly decreased childhood injury rates [6]. Similarly, Dubowitz et al. (2009) conducted a cohort study in the USA, showing that the SEEK model, an approach that stands for Safe Environment for Every Kid and integrates safety education into routine care, effectively prevented child maltreatment and injuries [11]. The SEEK model involves special training for residents, the use of a Parent Screening Questionnaire, and the engagement of a social worker to identify and address risk factors for child maltreatment, thereby promoting a safer environment for children.

Mental Health Screening

Mental health screening in primary care settings is vital for the early detection and management of psychological issues. The Committee on Psychosocial Aspects of Child and Family Health and Task Force on Mental Health (2009) conducted a systematic review emphasizing the importance of routine mental health assessments to identify conditions such as depression and anxiety early on [12]. They proposed competencies for pediatric primary care clinicians to prevent and address mental health and substance abuse problems, suggesting that collaborative relationships with mental health specialists and changes in the financing of mental healthcare are necessary for effective integration. Foy et al. (2010) found that incorporating mental health services within primary care settings improved access to care and reduced the stigma associated with seeking mental health support, enhancing overall well-being [13].

Oral Health Programs

Oral health is a crucial aspect of overall health, and preventive oral health programs have been shown to be effective in reducing dental caries and promoting oral hygiene. The Section on Pediatric Dentistry and Oral Health (2008) demonstrated that providing oral health education and fluoride treatments during pediatric visits significantly improved dental outcomes [6], reinforcing current concepts and scientific evidence supporting practice-based preventive oral health programs. Perrin et al. (2016) further showed that integrating oral health assessments into routine care decreased the prevalence of dental caries [7]. Additionally, Ramos-Gomez (2020) offers supplementary evidence on the benefits of early dental intervention and parental education, noting that children who experienced their first preventive dental visit by age 1 incurred lower dental-related costs [14].

Parental Education

Parental education plays a significant role in enhancing the effectiveness of preventive strategies. Wood and McDaniel (2020) highlighted that educating parents on child development, nutrition, and safety led to better health outcomes for children [8]. Their systematic review showed that parent education programs increased engagement and adherence to preventive measures. Shah et al. (2016) noted that integrating parental education into primary care visits improved the effectiveness of preventive strategies, enhancing health outcomes by increasing parental competence in accident prevention, reading behavior, and child-raising practices [15].

Detailed Summary of Findings

We provide a concise overview of the key findings from the included studies in a summary table (Table 1).

**Table 1:** Summary of Findings From the Included Studies SEEK: Safe Environment for Every Kid, ECEC: early childhood education and care

Reference	Study	Preventive Strategy	Study Type	Country	Key Findings	Conclusion
[1]	Alexander et al. (2017)	Immunization	Systematic review	USA	Enhanced screening and recognition of health risks	Multi-component interventions are effective
[2]	Brown and Perrin (2018)	Nutrition counseling	Systematic review	USA	Role of providers in dietary guidance	Effective in promoting healthy habits
[3]	Vine et al. (2013)	Physical activity programs	Review (narrative)	USA	Encouraged active lifestyles among children	Physical activity counseling is beneficial
[4]	Committee on Practice and Ambulatory Medicine (2000)	Developmental screening	Observational study	USA	Continuous monitoring during well-child visits	Crucial for early detection of developmental issues
[5]	Gorin et al. (2014)	Nutrition counseling	RCT	USA	Structured counseling during routine visits reduced childhood obesity	Structured counseling promotes healthy eating
[6]	Section on Pediatric Dentistry and Oral Health (2008)	Oral health programs	RCT	USA	Improved dental outcomes with education and fluoride treatments	Effective in preventing dental caries
[7]	Perrin et al. (2016)	Developmental screening	Observational study	USA	Early identification/intervention for developmental delays	Routine screenings improve cognitive outcomes
[8]	Wood and McDaniel (2020)	Parental education	Systematic review	USA	Better health outcomes with parent education	Increases parental engagement and adherence
[9]	Gavagan et al. (2010)	Immunization/preventive	Retrospective review	USA	No clinically significant difference in screening or immunizations between incentivized and non-incentivized clinics	Financial incentives may not significantly improve preventive care
[10]	Sherwood et al. (2013)	Physical activity programs	RCT	USA	Improved activity levels in at-risk children following primary care-based intervention	Effective for children at risk for obesity
[11]	Dubowitz et al. (2009)	Accident prevention	Cohort study	USA	Prevented child maltreatment with the SEEK model	Effective in integrating safety education
[12]	Committee on Psychosocial Aspects of Child and Family Health and Task Force on Mental Health (2009)	Mental health screening	Systematic review	USA	Early detection of depression/anxiety in pediatric settings	Routine assessments improve mental health outcomes
[13]	Foy et al. (2010)	Mental health screening	Systematic review	USA	Improved access to mental healthcare when integrated in primary care	Reduces stigma and enhances care access
[15]	Shah et al. (2016)	Parental education	Review	Hong Kong	Enhanced effectiveness of preventive strategies with parent education	Improves health outcomes for children
[16]	Hensrud (2000)	Clinical preventive medicine in primary care	Narrative/commentary	USA	Discusses rationale for and current implementation of preventive services in primary care	Highlights the importance of proactive preventive strategies
[17]	Hayek et al. (2023)	eHealth tools	Systematic review	USA	eHealth tools support best practices in nutrition/physical activity in ECEC	Potential for enhancing early childhood care environments
[18]	Sallis et al. (1998)	Healthful eating and activity	Review (narrative)	USA	Need for development/evaluation of youth nutrition and physical activity interventions	Justified public health benefits from interventions
[19]	dela Cruz et al. (2004)	Dental screening	Observational study	USA	Improved dental referral rates among at-risk children	Confidence/referral environment crucial for effective referrals
[20]	Savage (2004)	Infant oral healthcare	Cohort study	USA	Early dental intervention and parental education reduce early childhood caries	Comprehensive oral care programs crucial
[21]	Weber and Jenni (2012)	Pediatric screening	Review (narrative)	Germany	Parent counseling improves competence, accident prevention, and reading behavior	Scientific evidence supports pediatric screening

Risk of Bias Assessment

We assessed randomized controlled trials (RCTs) using the Cochrane Risk of Bias Tool, evaluating selection bias, performance bias, detection bias, attrition bias, and reporting bias. Observational studies (cohort or cross-sectional designs) were assessed using the Newcastle-Ottawa Scale, focusing on selection, comparability, and outcome assessment. For systematic reviews and narrative reviews, a formal tool (e.g., AMSTAR) was not specified in our original methods, so risk of bias judgments are more approximate.

After an independent assessment by two reviewers, any discrepancies were resolved through discussion. Studies were rated overall as having low, moderate, or high risk of bias (Table 2).

**Table 2:** Summary of Risk of Bias by Study RCT: randomized controlled trial, SEEK: Safe Environment for Every Kid

Reference	Study	Study Type	Overall Risk of Bias	Notes/Rationale
[1]	Alexander et al. (2017)	Systematic review	Moderate	Systematic methods reported; no detailed mention of registration
[2]	Brown and Perrin (2018)	Systematic review	Moderate	Broad search strategy; no explicit mention of AMSTAR-based bias assessment
[3]	Vine et al. (2013)	Review (narrative)	Moderate	Good summary; no standardized bias tool mentioned
[4]	Committee on Practice and Ambulatory Medicine (2000)	Observational study	Moderate	Continuous monitoring described; unclear if potential biases were mitigated
[5]	Gorin et al. (2014)	RCT	Moderate	Randomization described; unclear allocation concealment/blinding
[6]	Section on Pediatric Dentistry and Oral Health (2008)	RCT	Moderate	Intervention described; unclear if outcome assessors were blinded
[7]	Perrin et al. (2016)	Observational study	Moderate	Early detection outcomes well-described; potential confounders not controlled
[8]	Wood and McDaniel (2020)	Systematic review	Moderate	Preliminary investigation; no mention of protocol registration
[9]	Gavagan et al. (2010)	Retrospective review	Moderate	Retrospective design assessing pay-for-performance in community clinics; no major impact observed; potential confounding
[10]	Sherwood et al. (2013)	RCT	Moderate	Possible attrition bias if dropouts were not balanced or well-described
[11]	Dubowitz et al. (2009)	Cohort study	Moderate	Clear SEEK model; possible selection bias; limited blinding
[12]	Committee on Psychosocial Aspects of Child and Family Health and Task Force on Mental Health (2009)	Systematic review	Moderate	Formal bias assessment not clearly detailed
[13]	Foy et al. (2010)	Systematic review	Moderate	Methods for identifying included studies not extensively outlined
[15]	Shah et al. (2016)	Review (narrative)	Moderate	Summarized positive parenting; search strategy/inclusion criteria unclear
[16]	Hensrud (2000)	Narrative/commentary	Moderate	Broad overview of preventive medicine in primary care; limited methodological detail
[17]	Hayek et al. (2023)	Systematic review	Moderate	Potentially comprehensive search; limited detail on risk of bias in included studies
[18]	Sallis et al. (1998)	Review (narrative)	Moderate	Early review, limited methodology detail
[19]	dela Cruz et al. (2004)	Observational study	Moderate	Details on participant selection/confounders not fully explicit
[20]	Savage (2004)	Cohort study	Moderate	Clear outcomes, but possible unadjusted confounders/loss to follow-up
[21]	Weber and Jenni (2012)	Review (narrative)	Moderate	Methods for synthesizing evidence not clearly detailed

Table 2 presents the detailed risk of bias assessments for each included study, which collectively demonstrated an overall moderate risk of bias. This designation primarily reflects incomplete reporting of methodological details, occasional uncertainty about participant blinding, and variability in outcome measurement and analysis across studies. We found no direct evidence of selective outcome reporting, as primary endpoints were generally reported consistently. Using the GRADE approach, we rated the certainty of evidence for key preventive outcomes (e.g., immunization coverage and developmental screening accuracy) as moderate, underscoring generally consistent findings but acknowledging the methodological limitations and limited generalizability observed in the included studies.

Discussion

Principles, Relationships, and Generalizations

Our findings underscore the critical role of various preventive strategies in pediatric primary care, demonstrating their effectiveness in enhancing child health outcomes. The integration of these strategies into routine pediatric care is essential for preventing diseases, promoting healthy development, and addressing potential health issues early on.

Exceptions and Lack of Correlation

While the overall findings support the effectiveness of these preventive strategies, certain exceptions and lack of correlation must be noted. For instance, while immunization and developmental screening showed consistent positive outcomes, the impact of mental health screening and parental education varied across different studies. The Committee on Psychosocial Aspects of Child and Family Health and Task Force on Mental Health (2009) emphasized the importance of routine mental health assessments, yet some studies reported challenges in implementation due to stigma and resource constraints [12]. Similarly, parental education, although generally beneficial, showed variable effectiveness depending on the socioeconomic context and the level of engagement from parents [8,15].

Comparison With Previous Work

The effectiveness of developmental screenings is supported by findings from the Committee on Children With Disabilities (2001), which emphasized the importance of early developmental monitoring to improve cognitive and behavioral outcomes [22]. However, discrepancies exist in the literature regarding the sensitivity and specificity of various screening tools and their implementation across different clinical settings. For instance, Hirai et al. (2018) demonstrated significant state-level variation in developmental screening and surveillance rates among children, attributing this variability to differences in clinician training, screening frequency, healthcare settings, access to medical homes, and family socioeconomic status [23]. These findings suggest that, while early screening is valuable, its effectiveness is highly contingent on contextual factors. This underscores the need for standardized screening protocols and systems-level improvements to ensure consistent and reliable developmental assessments across diverse populations.

In the realm of nutrition counseling, Asmuniati et al. (2019) demonstrated that structured interventions significantly improve nutritional knowledge and energy intake among obese children, supporting our findings on the importance of nutritional guidance in primary care [24]. Similarly, Melnick et al. (2020) emphasized early nutrition education to increase preschool children's willingness to consume fruits and vegetables, aligning with our review's conclusions on the effectiveness of nutrition counseling [25].

Physical activity programs also show consistency with previous work. Kriemler et al. (2011) found that integrating physical activity initiatives into school and primary care settings enhances physical fitness and reduces obesity risk, which echoes our findings on the benefits of physical activity programs [26].

However, mental health screenings showed some discrepancies. While Talen et al. (2013) reported improved access and early identification of psychological issues through integrated mental health services in primary care, our review highlights the variability in outcomes and suggests that more targeted approaches are necessary to address barriers such as stigma and resource limitations [27]. This indicates a need for further research to develop effective mental health screening strategies in pediatric primary care.

Theoretical Implications

The theoretical implications of our findings are significant, suggesting that a multifaceted approach to preventive care in pediatrics is essential for optimizing health outcomes. The consistent success of immunization and developmental screenings points to the importance of early intervention and preventive measures in primary care settings. The mixed results for mental health screenings and parental education indicate a need for further research to develop more effective, context-specific strategies. These findings support a comprehensive model of pediatric care that integrates multiple preventive strategies, tailored to the specific needs and contexts of different populations.

Practical Applications

The practical applications of this review are wide-ranging. Healthcare providers can use these findings to enhance their preventive care practices by integrating successful strategies such as immunization, developmental screenings, and nutrition counseling into routine pediatric visits. Policymakers and healthcare administrators should consider supporting initiatives that address barriers to implementation, such as providing additional training for healthcare providers and ensuring adequate resources. Additionally, targeted interventions to improve mental health screenings and parental education could be developed, taking into account the socioeconomic and cultural contexts of different communities.

Limitations

This review is limited by potential language bias (only English publications were included) and resource constraints (only two reviewers performed screening and extraction). These factors may reduce the comprehensiveness of our findings.

## Conclusions

In conclusion, this systematic review underscores the importance of integrating multiple preventive strategies into routine pediatric primary care to enhance child health outcomes. Immunization, developmental screening, nutrition counseling, physical activity programs, accident prevention, mental health screening, oral health programs, and parental education each play a crucial role in promoting the well-being of children. Addressing barriers to implementation and tailoring strategies to specific contexts are essential for maximizing their effectiveness.
